# Combined replantation of the posterior arch of the atlas and bilateral axial lamina in the treatment of intradural schwannoma: a case report

**DOI:** 10.3389/fsurg.2024.1374208

**Published:** 2024-06-07

**Authors:** Ao Leng, Jiacheng Li, Lingzhi Meng, Qi Wang

**Affiliations:** Department of Orthopaedics, General Hospital of Northern Theater Command, Shenyang, China

**Keywords:** spinal cord neoplasms, cervical vertebrae, cervical atlas, surgery, case report

## Abstract

**Background:**

Laminotomy and laminar replantation have emerged as novel treatment modalities for intraspinal tumors, aiming to minimize postoperative complications and retain spinal mobility. However, existing research predominantly emphasizes their application in the thoracolumbar spine. The unique anatomy of the atlantoaxial segments necessitates surgical techniques that differ from those used in other spinal regions, and the clinical effect of such procedure remains unknown.

**Case presentation:**

A 61-year-old male patient with intradural schwannoma at the atlantoaxial level was operated on. The patient underwent posterior laminectomy, as well as a combined replantation of the posterior arch of the atlas and bilateral axial laminae. Postoperatively, the patient experienced significant neurological improvement, with no deformities or instability on the radiological assessments during the follow-up.

**Conclusion:**

Laminotomy with combined replantation of the posterior arch of the atlas and bilateral axial lamina emerges as an effective approach for managing intraspinal tumors at the atlantoaxial level. This technique not only offers ample operating space but also restores the stability of the spinal canal. Moreover, it preserves the mobility of the atlantoaxial segment, minimizes impact on adjacent segments, and mitigates the formation of postoperative fibrosis.

## 1 Introduction

Primary spinal cord tumors constitute 2%–4% of all central nervous system neoplasms (1). Annually, an estimated 3,000 new cases of primary spinal cord tumors are diagnosed in the United States, with an age-adjusted incidence rate of approximately 0.98 per 100,000 persons (2). Intradural extramedullary tumors constitute two-thirds of primary spinal cord tumors, which are generally benign neoplasms (3). Surgical excision is considered to be the treatment of choice, aiming to both completely remove the tumor and to reconstruct the stability of the spine. For tumors located in the upper cervical spine, the anatomical location and limited operating space present intractable challenges. Common procedures such as hemilaminectomy, bilateral laminectomy, and laminoplasty are frequently employed to achieve full tumor exposure. After tumor resection, pedicle screws are traditionally used to reconstruct spinal stability. While pedicle screw fixation offers robust stability, it may compromise mobility. This is particularly important at the atlantoaxial level since 50%–60% of rotation comes from the atlanto-axial articulation (4). Other drawbacks of pedicle screw instrumentation may include the risk of iatrogenic spinal stenosis, adjacent segment degeneration, and epidural fibrosis (5). In 1976, Raimondi first reported successful replantation of lamina in clinical treatment, showing promising surgical outcomes (6). Subsequently, some researchers began to employ laminar replantation for treating intraspinal tumors, but is mostly performed in the thoracolumbar spine (7).

In this report, we present a case involving an intradural schwannoma at the atlantoaxial level. The patient presented with progressive motor impairment and limb pain, along with decreased muscle strength in the right limbs, brisk biceps/triceps reflex (3+), and a positive Hoffman sign. A laminotomy and combined replantation of the posterior arch of the atlas and bilateral axial lamina were performed, and the patient experienced steady alleviation of symptoms during the follow-up.

## 2 Case description

A 61-year-old male patient presented with motor impairment and pain in the lower limbs and right arm, progressively over the last 4 years. His symptoms worsened during the last three months with new onset of subjective instability during deambulation and impaired fine movement in his right hand. The patient reported no positive findings in medical, family, and psycho-social history, and received no treatment previously. Physical examination revealed decreased muscle strength in the right limbs, brisk biceps/triceps reflex (3+), and positive Hoffman sign. Contrast-enhanced cervical Magnetic Resonance Imaging (MRI) revealed an irregular intradural-extramedullary mass measuring 3.7 × 1.7 × 3.0 cm, protruding outward through the left intervertebral foramen of C1-2. The lesion was of equal T1 and T2 signal with mild enhancement. The spinal cord was obvious and shifted to the right with altered signal on T2 weighted sequence, indicating a likely diagnosis of intradural-extramedullary schwannoma associated with myelopathy. Additionally, preoperative computed tomography angiography (CTA) of bilateral carotid arteries was performed to illustrate the trajectory of the carotid arteries and their positional relationship to the atlas and axis.

## 3 Diagnostic assessment

The patient underwent surgery with a posterior approach. A linear incision spanning from the inion to the C3 vertebrae spinous process was made. Subperiosteal dissection was performed to expose the posterior arch of the atlas and the bilateral laminae of the axis. The supraspinous ligament and interspinous ligament interspersed between C2 and C3 were dissected. Subsequently, the posterior arch of the atlas and bilateral laminae of the axis were interrupted with the ultrasonic osteotome (SoniMed) at approximately 2 mm from the medial edge of the vertebral canal. The head of the ultrasonic osteotome was sagittally inclined to the midline at approximately 20°. Afterwards, the entire posterior arch, bilateral axial lamina, and posterior ligamentous complex were excised, and pre-curved microplates were placed for readiness. After a median linear incision of the dura, the tumor was exposed and carefully removed without violating the tumor border. Macroscopically, the tumor displayed a gray-white to gray-red irregular shape, measuring 3.0 × 2.0 × 1.0 cm, enclosed in a complete surface capsule. Following tumor removal, the dura sac was sutured. The posterior arch of the atlas and bilateral axial lamina were repositioned and fixed in their physiological location. Interspinous ligament between C2 and C3 were sutured. Two drainage tubes were placed before the surgical incision was sutured layer by layer. The entire operation lasted for 2 h with a total blood loss of 100 ml. Postoperative histopathology confirmed the diagnosis of schwannoma with positive staining of Vimentin and S-100.

**Figure 1:**
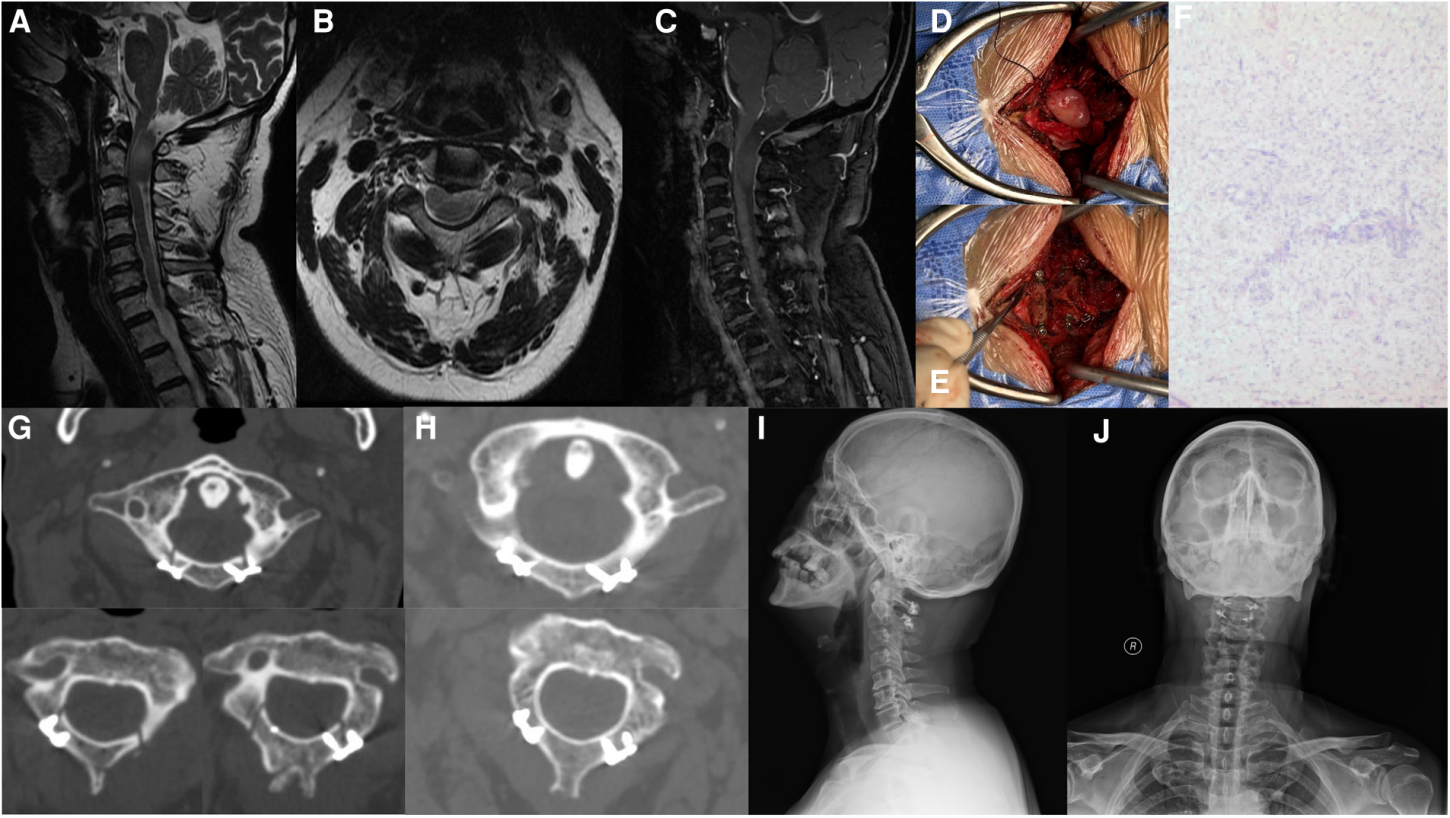
Case presentation. (**A**–**C**) Preoperative contrast-enhanced cervical Magnetic Resonance Imaging revealed an intradural-extramedullary mass measuring 3.7 × 1.7 × 3.0 cm, protruding outward through the left intervertebral foramen of C1-2. The lesion was of equal T1 and T2 signal with mild enhancement. (**D**) After removal of the posterior arch, bilateral axial lamina, and posterior ligamentous complex, a median linear incision of the dura was made to expose the tumor. (**E**) Following tumor removal, the posterior arch of the atlas and bilateral axial lamina were repositioned and fixed in their physiological location. (**F**) Postoperative pathology confirmed a diagnosis of Schwannoma. (**G**) Computed tomography (CT) conducted at 3 days after surgery showed satisfied position of the internal fixation. (**H**–**J**) Postoperative CT and plain radiograph revealed the progressive ossification of the posterior arch and axial lamina, with no signs of instability or deformities.

The postoperative recovery was characterized by a prompt improvement of the neurological deficits without any complications. The patient was discharged and advised to wear a neck collar for 3 months. Six months later, the patient was followed as an outpatient, and a nearly complete regression of symptoms was observed. Computerized tomography (CT) scan showed no signs of new instability and revealed the progressive ossification of the posterior arch and axial lamina. The 3-month postoperative visual analog scale score was 0 and the Neck disability index (NDI) was 8.0%. Spine plain radiographs performed 6 months after surgery confirmed the correct alignment of the laminae with no deformities (Figure 1).

## 4 Discussion

In cervical surgery, iatrogenic disruption of the posterior arch of the atlas and the axial lamina is occasionally performed, for traumatic, degenerative or neoplastic diseases. According to the “three-column theory”, the posterior column bears 24%–30% of the pressure and approximately 21%–54% of the rotational stress (8). Destruction of the posterior column structure affects the biomechanical stability of the spine, which may lead to delayed kyphosis and neurological damage. Consequently, posterior atlantoaxial fixation is commonly performed to reconstruct atlantoaxial stability. The posterior atlantoaxial fixation techniques include six main types: sublaminar wiring, interlaminar clamp, transarticular screw, screw-plate system, screw-rod system, and hook-screw system fixation (9, 10). Biomechanical studies have demonstrated that transarticular screw fixation provides the stiffest stabilization with the least amount of rotation and lateral bending, making it the “gold standard” for posterior atlantoaxial fusion. A fusion rate as high as 94.6% has been reported in literature (11). Meanwhile, screw-rod systems, especially the C1 pedicle/lateral mass screw combined with C2 pedicle/pars screw fixation, have become the most popular fixation techniques (9). However, posterior atlantoaxial fixation can be technically demanding and involves operation-related risks such as screw malposition and potential injury to the vertebral artery, spinal cord, and hypoglossal nerve. Other complications include the elimination of rotation at the atlantoaxial junction, accelerating degeneration in adjacent cervical segments, as well as the onset of severe neurological symptoms.

In 1976, Raimondi introduced lamina replantation for the first time (6). Although initially designed to preserve the normal architecture of the spine in patients who are still developing, limina replantation has now seen its use in a wider range of applications. Studies have reported its use in patients with intraspinal tumor or degenerative diseases such as ossification of the ligamentum flavum or lumbar disc herniation (12, 13). By retaining the natural posterior structures, lamina replantation restores stability and prevents epidural adhesion. Zhou et al. applied lamina replantation with ARCH plate fixation in 13 patients with thoracic and lumbar intraspinal tumors and observed a significant improvement in all patients (7). In a comparative study by Liu et al., 32 patients underwent tumor resection and lamina replantation while 26 patients underwent tumor resection and screw fixation. Compared with pedicle screw fixation, lamina replantation achieved similar symptom relief, along with shorter operation time, fewer postoperative complications, less impact on the mobility of the spine, and a lower incidence of adjacent segment degeneration (14). By retaining the spinous process ligament complex and maintaining the posterior tension, lamina replantation reduces the occurrence of kyphosis and iatrogenic spinal stenosis (15). Additionally, due to the fact that the soft tissue in the spinal canal is protected after the replantation of the lamina, the dural sac is separated from the posterior muscle to avoid epidural adhesion, preventing posterior tissue from protruding into the spinal canal. Moreover, lamina replantation causes less damage to muscular structure and articular movement, thus preserving the function of the spinal motion segments. This is particularly crucial in patients with fair functioning before surgery or adolescents who are still developing. Furthermore, considering the potential for tumor recurrence, revision surgery after reimplantation is much easier and safer than traditional surgery.

However, lamina replantation is predominantly performed in the thoracolumbar spine. To the best of our knowledge, there are no reports in the literature on the application of combined replantation of the posterior arch of the atlas and bilateral axial lamina. The atlantoaxial segment is a complex area with distinctive anatomy from other segments of the spine, necessitating different surgical techniques. Due to the anatomic variance of the carotid artery, a CTA examination is recommended before surgery to observe the trajectory of the carotid artery and avoid iatrogenic damage during the operation. During the laminotomy procedure, the insertion of a nerve dissector beneath the laminae assists the surgeon in identifying the medial surface of the pedicle. An ultrasonic osteotome is then used to create a grove on the lamina. The ultrasonic osteotome is sagittally tilted by about 10–20° to the midline, increasing the contact area and stability after replantation. Postoperatively, we recommend that the patient wear a cervical collar for 3 months, until x-ray shows complete healing across the osteotomy site. The period of stabilization could be prolonged if any risk factors for osteoporosis exist.

## 5 Conclusion

For intraspinal tumors at the atlantoaxial level, laminotomy and combined replantation of the posterior arch of the atlas and bilateral axial lamina can offer ample operating space and simultaneously reconstruct the stability of the spinal canal. This surgical approach effectively restores nerve function while preserving the mobility of the atlantoaxial segment, thereby minimizing the impact on adjacent segments and avoiding the development of postoperative fibrosis. However, a more extended follow-up with a larger population is essential to establish the long-term effectiveness of this procedure in preventing deformity and instability.

## Data Availability

The original contributions presented in the study are included in the article, further inquiries can be directed to the corresponding author.
